# Thymectomy in nonthymomatous myasthenia gravis - systematic review and meta-analysis

**DOI:** 10.1186/s13023-018-0837-z

**Published:** 2018-06-25

**Authors:** Antônio J. M. Cataneo, Gilmar Felisberto Jr., Daniele C. Cataneo

**Affiliations:** 1 Division of Thoracic Surgery, Department of Surgery, Botucatu School of Medicine, São Paulo State University, UNESP, Botucatu, SP CEP 18.618-970 Brazil; 2 Post-Graduation Program General Bases of Surgery, Botucatu School of Medicine, São Paulo State University, UNESP, São Paulo, Brazil

**Keywords:** Myasthenia gravis, Thymectomy, Drug therapy

## Abstract

**Background:**

The objective of this study is to evaluate by means of a systematic review, the efficacy of thymectomy as compared to medical treatment for non-thymomatous myasthenia gravis **(**MG).

**Methods:**

Medline, Embase, and Lilacs were searched for experimental and observational studies that compared non-surgical (drug therapy) and surgical treatment of non-thymomatous MG (thymectomy performed by the transsternal approach). Inclusion criteria were: studies that compared the two types of treatment and had at least 10 adult patients in each group. Exclusion criteria were articles published before 1970, as well as those that included patients treated before 1950. The outcomes evaluated were: remission, and improvement rates. RevMan 5.3 software provided by the Cochrane Collaboration was used. When the heterogeneity between the studies was greater than 75%, a meta-analysis was not performed according to RevMan guidelines.

**Results:**

The total number of patients evaluated in 19 articles selected was 5841 (2911 surgical and 2930 non-surgical). Two included randomized clinical trials showed superiority of the surgical treatment over the non-surgical. Four retrospective studies with 379 patients paired by gender, age, and other confounders, also showed superiority of surgical treatment (OR 4.10, 95% CI 2.25 to 7.44; I^2^ = 20%). In meta-analyses, remission assessed in 17 studies (5686 patients) was greater in patients who underwent surgical treatment (OR 2.34, 95% CI 1.79 to 3.05; I^2^ = 56%). For improvement assessed in 13 studies (3063 patients) were not appropriate to carry out the meta-analysis due to the high heterogeneity among the studies in the outcome (87%).

**Conclusion:**

Thymectomy may be considered effective in the treatment for non-thymomatous MG, with remission rate higher than for non-surgical treatment.

## Background

Myasthenia gravis (MG) is an autoimmune disease that affects the neuromuscular junction causing fluctuating weakness of skeletal muscles. It is considered a rare disease with an estimated prevalence of 7.77 per 100,000 [1]. The origin of autoimmune dysfunction in patients with MG is unknown, but thymic abnormalities and defects in immune regulation play important roles in patients with anti-AChR antibodies. The thymus is essential for T-cell differentiation and establishment of central tolerance [2, 3]. There are also genetic and hormonal components associated with the production of antibodies [4]. Drug therapy is carried out with medications that increase neuromuscular transmission, immunosuppressive drugs, plasmapheresis, immunoglobulins and monoclonal antibodies [5]. Surgical treatment is done by simple or extended thymectomy [6–8]. Although thymectomy has been used in the treatment of MG since 1941 [9], the role of thymectomy for MG is not completely understood. In a review published by Cochrane in 2013 [10], there were no studies included (empty review), and the authors’ conclusion is that there is insufficient evidence to support the use of thymectomy in non-thymomatous MG, and that randomized and quasi-randomized studies are needed. The Brazilian Department of Health approved in 2015 a protocol of therapeutic guidelines in MG [11], where the role of thymectomy in patients with MG without the presence of thymoma is considered uncertain. This concept was based on a review published in the year 2000 [12] with 21 retrospective studies published from 1953 to 1998; the participating patients had been accrued since 1932. In 2016 the American Academy of Neurology [13] published “International consensus guidance for management of myasthenia gravis”: “In non-thymomatous MG, thymectomy is performed as an option to potentially avoid or minimize the dose or duration of immunotherapy or if patients fail to respond to an initial trial of immunotherapy or have intolerable side-effects from that therapy”. This shows that until recently thymectomy in non-thymomatous MG was uncertain or optional. In 2016 a randomized clinical trial (RCT) showed that thymectomy improved clinical outcomes over a 3-year period in patients with non-thymomatous MG [14]. Do we now have sufficient evidence to place thymectomy together with the other treatments recommended for non-thymomatous MG?

Therefore, the objective of our study is to evaluate the efficacy of surgical treatment (thymectomy) as compared to non-surgical in non-thymomatous MG, conducting a systematic review of experimental or observational studies.

## Materials and methods

### Criteria used to consider studies for this review

#### Studies

Randomized clinical trials (RCTs) or non-randomized controlled studies or observational studies (with at least 10 patients undergoing each intervention), comparing medical management (any type) with surgical treatment (thymectomy performed by the transsternal approach) to treat generalized MG in patients without thymoma. We excluded studies that analyzed only one intervention. We excluded articles published before 1970, as well as those that included patients treated before 1950, due to advances in anesthesiology, surgical techniques, and the launch of prednisone in the second half of the twentieth century.

#### Participants

Adults with generalized non-thymomatous MG.

#### Interventions

Any type of medical management for MG compared with surgical treatment. Medical management could include any type of drugs, and surgical management could include simple or extended thymectomy.

#### Outcomes

Remission rates (asymptomatic without medication) and improvement rates (reducing medication or asymptomatic with medication).

### Search methods for studies identification

#### Electronic searches

Pubmed (1966 to December 2016); Embase (1980 to December 2016); Lilacs (www.bireme.br/) (1982 to December 2016); www.clinicaltrials.gov (assessed December 2016).

A comprehensive search strategy was used: (Generalized Myasthenia Gravis), and (Thymectomies or thymectomy), and (Drug Therapy or Drug Therapies or Chemotherapy or Chemotherapies or Pharmacotherapy or Pharmacotherapies). The search strategy was adapted for each database in order to achieve more sensitivity. There was no restriction concerning language or publication status**.** The references of relevant publications found by the search were screened for further studies and experts in the field were also contacted.

### Data collection and analysis

#### Studies selection

Two review authors (GFJ and AJMC) independently examined titles and abstracts in order to remove irrelevant reports; retrieved full-text copies of the potentially relevant reports; identified multiple reports from the same study by checking authors’ names, location and setting, details of the intervention, date and duration of the study; examined full-text reports for compliance with eligibility criteria; if necessary corresponded with authors in order to clarify any questions related to the study; made a final decision on study inclusion.

#### Data extraction and management

Two review authors (GFJ, DCC) independently extracted data from eligible studies and summarized them using a data extraction form. This summary contained the baseline characteristics of the study and included study type, age, gender, type of treatment, total number of participants, number of patients in each arm, follow-up, interventions and outcomes assessed, as well as initial and end classification of MG. In addition, duration of follow-up and numbers lost to follow-up were extracted. Where one same study gave origin to more than one publication, data were extracted from all relevant publications but not duplicated.

Assessment of risk of bias in studies included**:** in RCTs, two review authors (GFJ, DCC) assessed each trial independently, and assessed study quality by using the ‘Risk of bias’ tool for Cochrane reviews [15]. In observational studies risk assessment for bias was not done due to lack of consensus for the application of this assessment in these studies, but they were considered as biased and subject to the effect of confounders.

Disagreement was solved by consensus with participation of all authors.

#### Measures of treatment effect

The outcomes were treated as dichotomous variables and presented as odds ratio with corresponding 95% CIs.

#### Investigation of heterogeneity (I^2^)

In order to quantify the inconsistencies of the studies included in the meta-analysis, the heterogeneity test (I^2^) was used [16]. Presence of substantial heterogeneity was considered when I^2^ > 75%; in this case the combination should be considered inappropriate, and the results should be presented in narrative form.

#### Data synthesis

Data was analyzed using RevMan 5.3 software provided by Cochrane Collaboration. If the I^2^ value was greater than zero, a random-effects model was applied.

## Results

### Description of studies

#### Results of the search

**Fig. 1:**
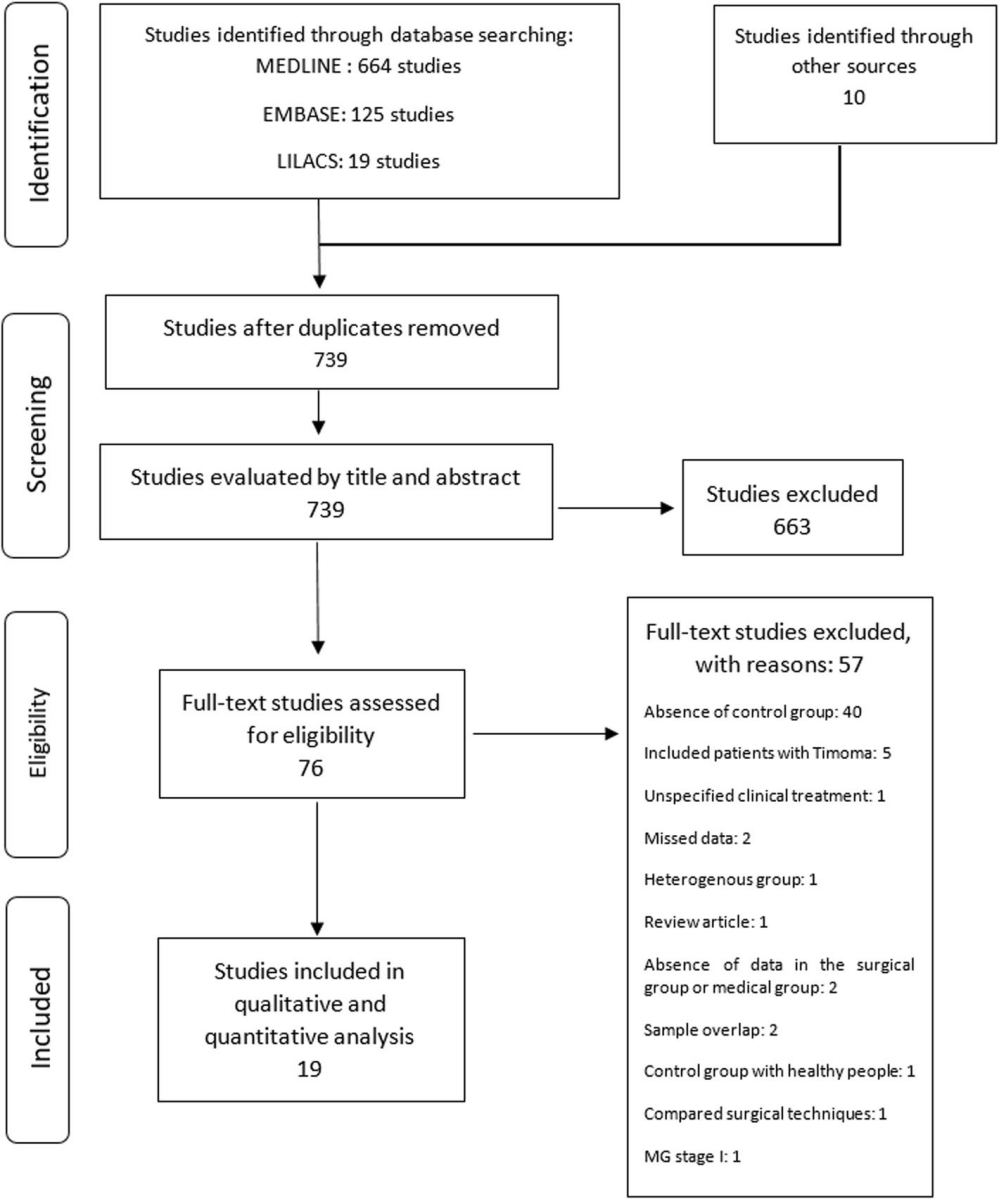
Flowchart showing the studies identified and evaluated during the review

The search conducted in December 2016 recovered 664 studies in Medline, 125 in Embase, 19 in Lilacs and 10 through other sources. After exclusion of duplicate and analysis of titles and abstracts, 76 articles were selected and obtained in full paper copies. Of these, 19 publications were selected for this review [14, 17–34] (Fig. 1).

#### Studies included

The main characteristics of the selected studies were summarized in Table 1. The total number of patients evaluated in the 19 articles selected was 5841 with 2911 belonging to the surgical group and 2930 to the non-surgical group. Only two studies were RCTs [14, 23], all others were observational (case-control). But four of them were matched by sex, age, time to diagnosis, age at the beginning of the disease, duration of the disease, and follow-up time. A separate meta-analysis of these four studies was performed.

**Table 1:** Demographic data of studies selected for this review

Author/Year (period) country	Study design	Surgical total (female, male)	Medical total (female, male)	Remission		Improvement		Follow-up	Medical treatment
				Surgical	Medical	Surgical	Medical		
Wolfe/2016 [14] (2006–2012) 36 centers, 32 in USA	Multicenter RCT	66 (50,16)	60 (39,21)			Average MG score 6.15	Average MG score 8.99	3 years	Predinisone
Barnett/2014 [17] (2000–2013) Canada	Single center Retrospective Unmatched (U) matched (M)	U- 183 (124,59) M- 49 (25, 24)	U- 212 (89, 123) M- 49 (27, 22)	R/MM^a^ U-40 (22%) M^−10^(21%)	R/MM^a^ U-49 (23%) M^− 3^(06%)			60 months	Prednisone
Bachmann/2009 [18] (1980–2005) Germany	Single center Retrospective	84 (56, 28)	88 (50, 38)	35 (42%)	12 (14%)	32 (38%)	15 (17%)	Mean = 10 years	Pyridostigmine/azathioprine/glucocorticoids/ plasmapheresis
Al-Moalen/2008 [19] (1984–2006) Saudi Arabia	Single center Retrospective	73 (49, 24)	23 (13, 10)	10 (14%)	0	24 (33%)	6 (26%)	Mean = 7.2 years	Pyridostigmine/prednisolone/azathioprine
Tsinzerling/2007 [20] (1956–2006) Sweden	Single center Retrospective	261 (179,82)	211 (105,106)	77 (29.5%)	32 (15%)	139 (53%)	142 (67%)	> 1.5 year	Azathioprine/cyclosporine/steroids
Kawaguchi/2007 [21] (1999–2000) Japan	Multicenter Retrospective	20 (10, 10)	14 (11, 3)	6 (30%)	3 (21%)	10 (50%)	8 (57%)	11.7 years (surgical) 7.8 years (non-surgical)	Cholinesterase inhibitors/corticosteroids/other immunosuppressive agents
Werneck/2000 [22] (1973–1995) Brazil	Single center Retrospective matched	28 (20, 8)	28 (20, 8)	6 (21%)	9 (32%)	8 (29%)	1 (4%)	Mean Surgical = 6.3 years Non-surgical = 3.5 years	Prednisolone/ cholinesterase Inhibitors/ immunosuppressive agents
Lorenzana/1999 [23] (1991–1995) Colombia	Single center RCT	11 (10, 1)	18 (16, 2)	Fatigue gain 9.1 s.	Fatigue gain 2.2 s.	Strength 2.1	Strength 0.25	2 years	Prednisolone/pyridostigmine/prostigmine
Robertson/1998 [24] (1965–1997) UK	Single center Retrospective	22 (21,1)	41 (27,14)	6 (27%)	3 (7%)	12 (55%)	15 (37%)	Not reported	Anticholinesterase/steroids/azathioprine/plasmapheresis
Evoli/1998 [25] (not informed) Italy	Single center Retrospective matched	45 (35,10)	20 (14,6)	15 (33%)	2 (10%)	Not reported	Not reported	≥ 3 years	cholinesterase Inhibitors/ immunosuppressive Agents/corticosteroids
Mantegazza/1990 [26] (not informed) Italy	Multicenter Retrospective	555 (not reported)	313 (not reported)	84 (15%)	19 (6%)	160 (29%)	56 (18%)	Mean = 4,9 years	Glucocorticoids/imunossupressive agents/ plasmapheresis
Donaldson/1990 [27] (1975–1988) USA	Single center Retrospective	90	57	23 (26%)	5 (9%)	30 (33%)	11 (19%)	Mean 8 years	Prednisolone/pyridostigmine/azathioprine/cyclophosphamide/ plasmapheresis
Valli/1987 [28] (1973–1985) Italy	Single center Retrospective	63	28	22 (35%)	2 (7%)	28 (44%)	19 (68%)	2–12 years	Cholinesterase inhibitors/corticosteroids/azathioprine
Papatestas/1987 [29] (1951–1985) USA	Single center Retrospective	788 (not reported)	1048 (not reported)	181 (23%)	146 (14%)	Not reported	Not reported	Not reported	Not reported
Assis/1987 [30] (not informed) Brazil	Single center Retrospective	19 (17, 2)	14 (12, 2)	6 (32%)	3 (21%)	12 (63%)	10 (71%)	8–24 years	Anticolinesterasic
Oosterhius/1981 [31] (1960–1980) The Netherlands	Single center Retrospective	144	183	39 (27%)	34 (19%)	Not reported	Not reported	10.2 years (surgical) 16.8 years (non-surgical)	Cholinesterase inhibitors/corticosteroids/azathioprine
Emeryk/1976 [32] (1963–1973) Poland	Single center Retrospective	112 (90,22)	75 (52,23)	26 (23%)	7 (9%)	70 (62%)	35 (47%)	Mean 3 years	Cholinesterase inhibitors
Buckingham/1976 [33] (?-65) USA	Single center Retrospective matched	80 (64,16)	80 (64,16)	27 (34%)	6 (7.5%)	26 (32%)	13 (16%)	19.5 years (surgical) 23 years (non-surgical)	Not reported
Perlo/1971 [34] (?) USA	Multicenter Retrospective	267 (217,50)	417 (?)	92 (34%)	71 (17%)	109 (41%)	46 (11%)	1–28 years	Edrophonium chloride/neostigmine
Total		2911	2930						

^a^R/MM- Remission or minimal manifestation

#### Types of intervention

Surgical treatment: simple or extended thymectomy.

Non-surgical treatment: anticholinesterasic drugs (prostigmine, pyridostigmine), immunosuppressive agents (azathioprine), plasmapheresis, corticosteroids.

#### Types of outcomes

Seventeen studies assessed remission, 13 assessed improvement. One assessed jointly remission and minimal manifestation [17].

#### Studies excluded

The 57 studies excluded and the reasons for exclusion are depicted in Fig. 1.

### Risk of bias in included studies

Only two RCTs were found, one of them used the time-weighted average Quantitative MG score as the outcome, and randomized the patients to thymectomy + prednisone or only prednisone [14]. The risk of bias for this study was considered low, since randomization was done by a computer program, with the professionals as well as the outcomes assessors blind to the procedure. Another RCT [23] was considered as with moderate risk of bias, because although the selection was random, there was no blindness of the outcome assessors, since the surgical scar was visible. The other studies were case series or case-control, therefore subject to the effect of confounders. Four case-control studies matched the patients by age, gender, and other confounders, thus reducing the risk of bias [17, 22, 25, 33].

### Descriptive analysis of the studies

1. Wolfe et al. [14], compared the surgical and clinical treatment of MG by conducting a multicenter RCT (36 centers, 32 in USA). A total of 126 patients between 2006 and 2012 were enrolled with 66 patients in the surgical group (thymectomy plus prednisone) and 60 in the non-surgical group (prednisone alone). Patients 18 to 65 years of age with disease duration of less than 5 years, Myasthenia Gravis Foundation of America (MGFA) clinical class II to IV and elevated circulating concentrations of acetylcholine-receptor antibody were included. The primary outcomes were the time-weighted average Quantitative MG score and the average dose of prednisone required over a period of 3 years. The surgical group had a lower time-weighted average Quantitative MG score than the non-surgical group (6.15 vs. 8.99, *p* < 0.001). The average dose of prednisone required was also lower in the surgical group as compared to the non-surgical group (44 mg vs. 60 mg, *p* < 0.001). Furthermore, in the thymectomy patients, the use of azathioprine (17% vs. 48% *p* < 0.001), and hospital admission for exacerbations (9% vs. 37%, *p* < 0.001) were lower.
2. Barnett et al. [17], in a retrospective single center study in Canada analyzed the efficacy of thymectomy in achieving remission or minimal manifestation (R/MM) status in patients with non-thymomatous MG. The primary outcome was the hazard ratio (HR) for achieving R/MM status at the last visit, fitting a Cox model on the matched dataset. Data analysis was made with two groups of the cohort according to the subjects data, one unmatched (*n* = 395), one matched (*n* = 98) by age, gender, time to diagnosis, MGFA status at diagnosis (I, II, III, and IV-V), and use of prednisone, azathioprine and mycophenolate mofetil during the follow-up period. The results of the analysis of the matched cohort group were: patients with thymectomy (*n* = 49) had a higher likelihood than controls (*n* = 49) of achieving R/MM with time (HR 1.9, 95% CI 1.6 to 2.3). The estimated rates of R/MM at 5 years were 21% (95% CI 16 to 40) for the thymectomy group, and 6% (95% CI 0 to 13) for controls, and there was an absolute estimated difference of 15% (95% CI 1 to 29), with a number needed to treat (NNT) = 7.
3. Bachmann et al. [18] in a retrospective single center study in Germany, reported a series of patients with MG. Patients were seen in the outpatient clinic, where a modified Osserman score and quality of life score were evaluated at the end of the follow-up period for all surviving patients. A total of 172 patients with MG (modified Osserman score 1–4) were followed after thymectomy (*n* = 84) or conservative treatment (*n* = 88) for a median time of 9.8 years. Eleven patients with generalized MG underwent conservative treatment due to older age. Patients who underwent thymectomy had significantly greater rates of remission and improvement as compared to those who had conservative treatment. Furthermore, they had a significantly greater survival (mortality MG-related: 10 in conservative treatment X 0 in thymectomy patients). The patients who underwent thymectomy had a higher modified Osserman score as compared to those who had conservative treatment (2.6 ± 0.7 vs 2.0 ± 0.9; *P* = 0.004), while no significant differences were found concerning age and gender.
4. Al-Moallem et al. [19] in a single center in Saudi Arabia retrospectively studied 104 patients (96 without thymoma and 8 with thymoma) followed over a mean period of 7.2 years (range 1 to 22 years). Patients who had thymectomy (non-thymomatous, *n* = 73) were compared to those who were not operated (*n* = 23) in relation to disease outcomes according to the MGFA post-intervention status criteria. Age at onset was 22.5 ± 9.3 years in females and 28.2 ± 15.9 years in males. At diagnosis, a majority of patients had moderate generalized weakness, equivalent to MGFA class III severity. Ten patients after thymectomy and none of the non-surgical patients had achieved complete stable remission (*p* = 0.111), four surgical and none non-surgical had pharmacological remission. Only patients who had thymectomy and no thymoma achieved any remission (14 surgical X 0 non-surgical, *p* = 0.02).
5. Tsinzerling et al. [20] in a retrospective single center study in Sweden reported 537 patients of whom 326 were thymectomised. Follow-up time was 1.5–50 years. Thymoma was found in 65, hyperplasia in 185 and a normal thymus in 76 patients. The trans-sternal surgical approach for thymectomy was used in 255 patients (78%). In five patients with thymoma, MG appeared after thymectomy. In the 261 patients operated without thymoma there was remission in 29.5%, whereas in the non-operated patients the remission was 15%.
6. Kawaguchi et al. [21] studied retrospectively 34 patients (20 post thymectomy and 14 non-surgical) recruited from 19 medical centers in Japan in order to investigate whether thymectomy is beneficial for late-onset (> 50 years) MG patients with no thymoma, particularly for those with mild generalized weakness. MGFA scores before treatment were in patients with thymectomy (50% < III and 50% ≥ III) and in non-operated patients (86% < III and 14% ≥ III). At the end of follow-up six patients who underwent thymectomy and three who were not operated had remission. MGFA score II was found in 10 surgical and in eight non-surgical, MGFA score ≥ III in none surgical and in two non-surgical. In 22 patients (10 post thymectomy and 12 non-surgical) MGFA score II before treatment, MGFA clinical classes at the end of follow-up were respectively in surgical and non-surgical patients: no symptoms 5(50%) X 2(16.7%), class I 2(20%) X 1(8.3%), class II 3(30%) X 7(58.3%), class III 0(0%) X 2(16.7%). Classes > II were observed in 3 out of 10 surgical patients and in 9 out of 12 non-surgical patients (*p* = 0.045).
7. Werneck et al. [22], in a retrospective study, in a single center in Brazil, paired 28 patients submitted to thymectomy with 28 patients with no surgical treatment. The pairing was done for gender, age of onset and duration of disease, in order to compare different parameters in similar populations. These groups were statistically similar in regard to the initial Osserman scale, use of corticosteroid and anticholinesterase. In the retrospective evaluation, it was considered remission when the patient was asymptomatic without medication, and improved when there was a decrease of 1 or more in the functional scale. There were remissions in 6 surgical and 9 non-surgical patients, and improvement in 8 surgical and 1 non-surgical patients.
8. Lorenzana et al. [23], in a RCT performed at a single center in Colombia, analyzed the results by comparing muscle strength and fatigue measured at intervals that varied from 3 months to 24 months; patients aged 15–50 years with illness duration of less than 5 years. In the surgical group (*n* = 11) strength improved 2.1 in the strength scale, statistically significant (95% CI 0.86 to 3.35; ρ = 0.004), while in the non-surgical group (*n* = 18) the improvement was 0.25 (95% CI 0.80 to 1.30; ρ = 0.612). For fatigue, the non-surgical group had an average gain of 2.2 s (95% CI 0.81 to 5.2; *p* = 0.138), and the surgical group had average gain of 9.1 s (95% CI 0.37 to 17.82; *p* = 0.043).
9. Robertson et al. [24] in an epidemiological study in Cambridgeshire, England reported 100 cases of MG in a population of 684,000. Thirty four underwent thymectomy, 12 with thymoma. Fourty one with generalized disease and 25 with ocular form were not operated. Remission rate in 22 surgical patients without thymoma was 27%, and in 41 generalized disease non-surgical patients the remission was 7%.
10. Evoli et al. [25] (abstract) a single center matched retrospective study in Italy. Patients were included in the study according to the following criteria: generalized MG with onset up to the age of 45 years, detectable serum anti-acetylcholine receptor antibodies, absence of thymoma, minimum follow-up of 3 years. Twenty patients had not been thymectomized having refused surgery: they were matched with thymectomized patients by gender, age at MG onset (±2 years), disease severity and follow-up duration. Non-thymectomized patients were 6 males and 14 females with age at MG onset ranging from 17 to 45 years (mean 30.7 ± 10.2) and mean follow-up of 13.6 ± 11.4 years. Thymectomized patients were 45 (35 females: 10 males) with a mean age at onset of 26 ± 9.2 and mean follow-up of 15.3 ± 4 years. With respect to MG severity, 10/20 non-thymectomized and 28/45 thymectomized patients had mild disease. Remission rate was 10% in the non-thymectomized and 33.3% in the thymectomized group.
11. Mantegazza et al. [26], a multicenter (6 centers) retrospective study in Italy, analyzed 555 patients submitted to thymectomy and 313 non-surgical patients and found remission in 84 surgical and 19 non-surgical (remission was considered when the patient was asymptomatic without medication for at least one year). In 160 surgical and in 56 non-surgical patients there were pharmacological remissions (patients remained asymptomatic with medication).
12. Donaldson et al. [27], a single center retrospective study in USA from 1975 to 1988. Ninety patients without thymoma underwent thymectomy and 57 received medical treatment. Remission occurred in 26% of the surgical group and in 9% of the non-surgical group.
13. Valli et al. [28], a single center retrospective study in Italy, analyzed 63 patients without thymoma submitted to thymectomy and 28 non-surgical patients. Remission occurred in 35% of the surgical group and in 7% of the non-surgical group.
14. Papatestas et al. [29], conducted a retrospective study at a single center in the USA from 1951 to 1985. Thymectomy was performed in 788 patients and 1048 were medically treated. Remission occurred in 181 patients (23%) of the surgical group and in 146 patients (14%) of the non-surgical group.
15. Assis et al. [30], conducted a retrospective study at a single center in Brazil with follow-up of 8 to 24 years. Nineteen patients underwent thymectomy and 14 received medical treatment. There were 6 remissions in the surgical group and 3 in the non-surgical group. Improvement occurred in 12 of the surgical group and in 10 of the non-surgical group.
16. Oosterhuis, 1981 [31], conducted a retrospective study at a single center in the Netherlands from 1960 to 1980. One hundred fourty four patients underwent thymectomy and 183 received medical treatment. Remission occurred in 27% of the surgical group and in 19% of the non-surgical group.
17. Emeryk and Strugalska, 1976 [32], conducted a retrospective study at a single center in Poland from 1963 to 1973. One hundred twelve patients without thymoma underwent thymectomy and 75 received medical treatment. Remission occurred in 23% of the surgical group and in 9% of the non-surgical group.
18. Buckingham et al. [33] conducted in a single center in USA a computer-assisted retrospective matched study. Of 563 MG patients without thymoma up to 1965, 104 had thymectomy. Each surgical patient was matched with medical patient on basis of age, sex and severity and duration of disease. Eighty of the 104 surgical patients could be matched satisfactorily with 80 non-surgical. Remission occurred in 34% of the surgical group and in 7.5% of the non-surgical group.
19. Perlo et al. [34] conducted in a multicenter retrospective study in USA. Two hundred sixty seven patients without thymoma underwent thymectomy and 417 received medical treatment. Remission occurred in 34% of the surgical group and in 17% of the non-surgical group.

### Effects of interventions (meta-analysis)

#### Remission

**Fig. 2:**
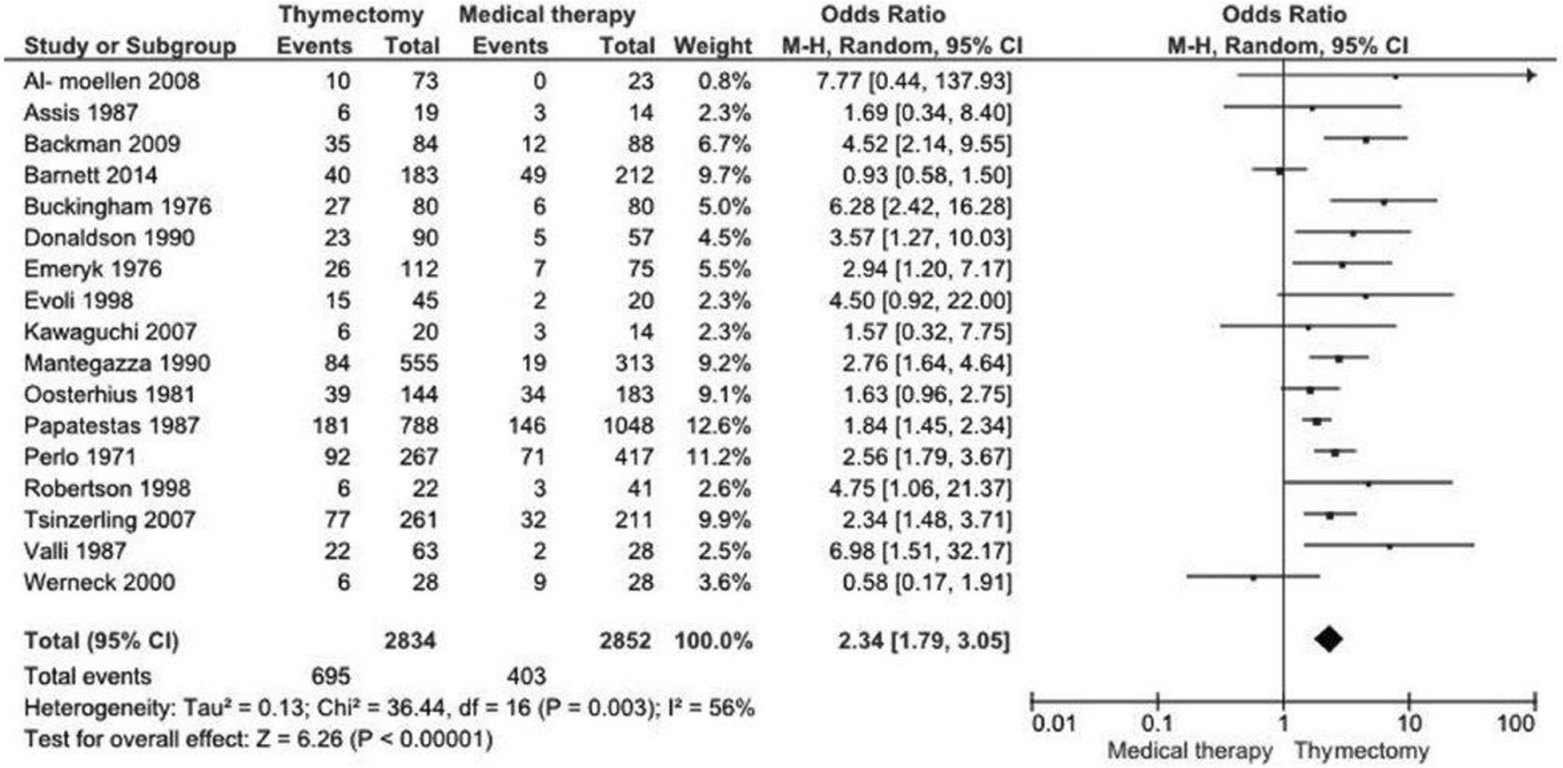
Meta-analysis of the remission in 17 studies (OR 2.34, 95% CI 1.79 to 3.05; I^2^ = 56%). The chance of remission in the thymectomy group is 2.34 times greater than in the medical group

The analysis of remission was done in 17 studies [17–22, 24–34] including 5686 patients. One study [23] presented this result in a qualitative and quantitative manner, and the most important and recent study presented time-weighted average Quantitative MG score after treatment [14], therefore combination with the other studies was not possible. There was a difference between thymectomy and non-surgical treatment (OR 2.34, 95% CI 1.79 to 3.05; I^2^ = 56%, Fig. 2). The chance of remission in the thymectomy group is 2.34 times greater than in the medical group.

#### Improvement

**Fig. 3:**
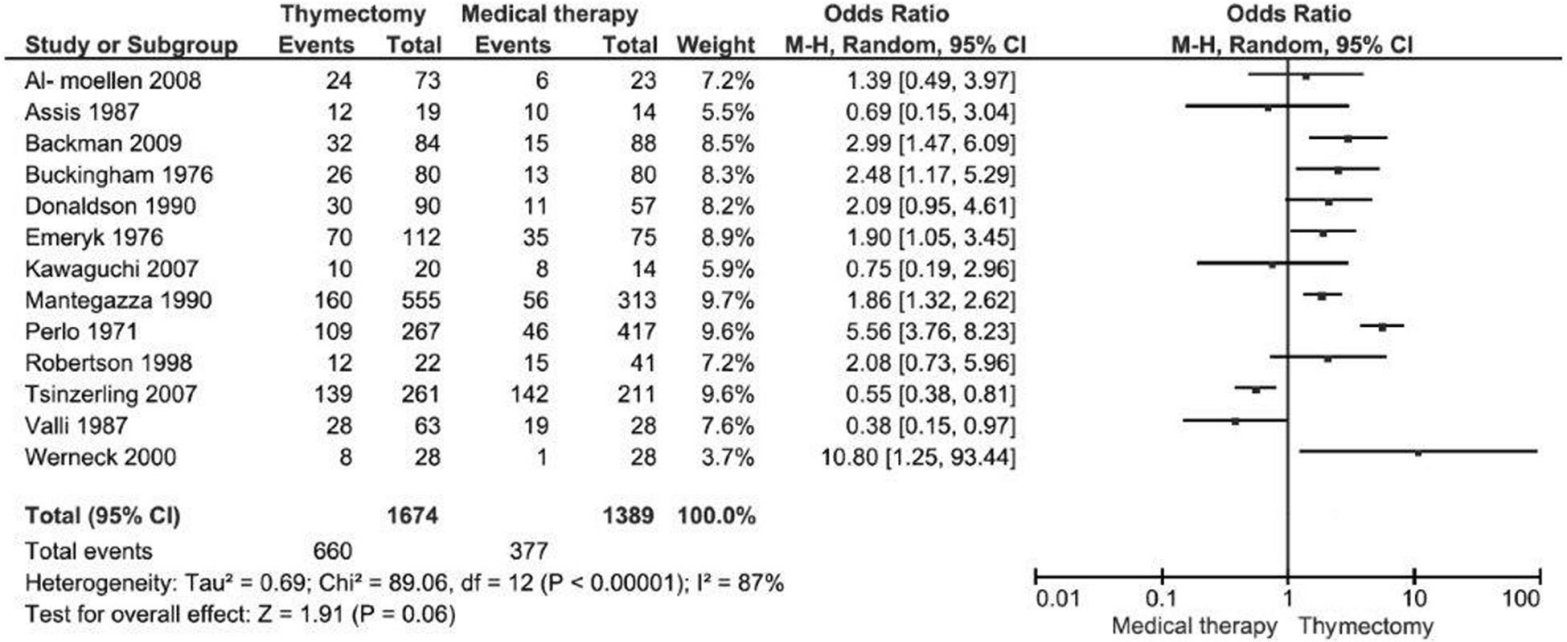
Analysis of the improvement in 13 studies. It was not appropriate to perform the meta-analysis because of high heterogeneity (87%)

The improvement analysis could be conducted only in 13 studies [18–22, 24, 26–28, 30, 32–34], including 3063 patients, but due to the high heterogeneity between the studies (87%) in this outcome, it was not appropriate to conduct the meta-analysis (Fig. 3).

#### Remission + improvement in matched studies

**Fig. 4:**
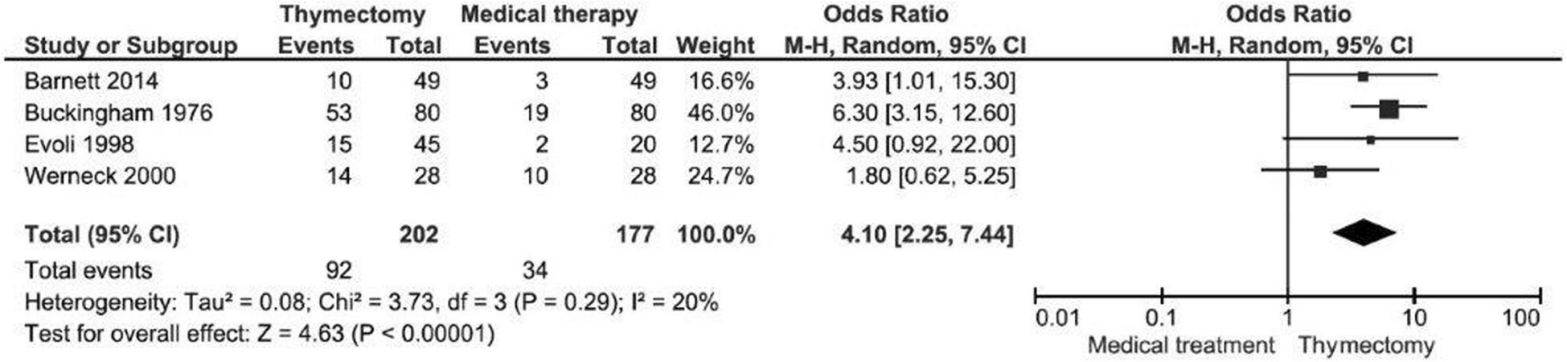
Meta-analysis of the remission + improvement in matched studies (OR 4.10, 95% CI 2.25 to 7.44; I^2^ = 20%). The chance of remission or improvement in the thymectomy group is four times greater than in the medical group

Meta-analysis was done with four studies that matched 379 patients by gender, age, and other confounders [17, 22, 25, 33]. The meta-analysis was performed with the remission added to the improvement. The thymectomy had a four times greater chance than the medical treatment of remission or improvement of MG (OR 4.10, 95% CI 2.25 to 7.44; I^2^ = 20%, Fig. 4).

## Discussion

Thymectomy for the treatment of MG is not completely understood. As the disease has a relatively low incidence and different etiologies, the construction of homogeneous groups in enough number is quite difficult. We included nineteen studies in this review, with a total of 5841 participants, but most of them are observational studies, and some bringing patients treated 50 years ago. The clinical heterogeneity of the observational studies coupled with the heterogeneity between the epochs in which the studies were carried out leads us to reduce the quality of evidence in the meta-analysis. Therefore, although this review has shown that MG’s chance of remission with thymectomy is greater than with drug therapy, this result should be viewed with caution, but thymectomy cannot be ignored.

The services that address these patients have their own preferences between medical or surgical treatment, what impairs the performance of randomized studies. The largest RCT comparing thymectomy with medical treatment, although multicentered, with 36 centers involved, was unable to reach the 200 patients foreseen in the initial protocol, having to increase the age group from 60 to 65 years and increase the duration of the disease from < 3 to < 5 years [14]. This shows the difficulty in conducting RCT when we want to test a surgical intervention, especially in diseases of low prevalence. This requires us to accept observational studies in decision making, and also to perform systematic reviews with or without meta-analysis [35]. Most of the studies included in this review were case-control, being considered of high risk of bias, but these studies reinforce the findings of the two RCTs found in the literature, one of them considered to have a low risk of bias and fairly large follow-up [14].

The inclusion of observational studies in systematic reviews has already been done by Cochrane [36]. Some particularities of observational studies may reduce the risk of bias. If the control group is selected from the same population of cases, there is low selection bias. However, in the observational studies included there is no reference to whether the control group was selected from the same population from which the cases were selected; four studies matched patients with the same characteristics [17, 22, 25, 33]. The performance bias is high, because there was no masking of participants and professionals, and there was certainly a selection bias when the authors opted for surgical treatment or medical treatment. This bias is reduced in the studies that matched the participants according to the confounding elements. The detection bias is low, since the outcomes are taken from medical records; therefore those assessing the outcomes are independent.

Meta-analysis of observational studies provide support for clinical practice until RCTs are conducted, although clinical and methodological heterogeneities are observed due to the nature of observational studies [35]. Possibly the sources of heterogeneity beyond the clinical differences between observational studies were also methodological, since the studies were retrospective. Nevertheless, there are some RCTs, therefore observational studies may serve to reinforce the evidence demonstrated by RCTs. The difficulties in conducting randomized studies of surgical treatments have already been discussed in the literature. They include: unwillingness to use randomization on the part of patients and surgeons; different levels of surgical ability and expertise to do different techniques; inability to “blind” evaluators to the interventions, since these are visible; inability to force randomization. However, if we do not consider the observational studies, we leave aside a significant amount of information reported in the literature that would be useful for both clinicians and the patients seeking information on the best treatment for the disease.

The superiority of surgical treatment over medical treatment in disease remission varied widely among the studies included in this review, from a non-benefit [22] to a large benefit [33].

Of the 13 studies that evaluated improvement, only six in the meta-analysis showed superiority of surgery over medical treatment [19, 22, 26, 32–34], but none of them showed superiority of the conservative treatment. Most of the studies, individually, demonstrated no difference between the two types of intervention for this outcome. This is due to the small number of patients in each study. The meta-analysis for outcome improvement was not performed because of high heterogeneity among studies for this outcome (87%), which makes improper the combination of these studies. In a RCT that analyzed the MG score and the necessary dose of prednisone, there was a better score and less amount of prednisone required in the surgical group [14].

Unfortunately only four studies matched patients with the same characteristics to compare the two interventions. Certainly, with the pairing, each study will analyze a smaller number of patients, but we would have a reduction in the risk of bias, and more reliable evidence in the meta-analysis of the retrospective studies.

In the last review comparing conservative treatment with thymectomy, published in 2016 [37], the authors analyzed 27 studies, and concluded that thymectomy is superior to conservative treatment with only medication in remission of MG. In this review the authors included four studies published before 1970, and three studies that included patients treated before 1950. In our review we excluded these studies due to advances in anesthesiology, surgical techniques and the introduction of prednisone that occurred after 1950. We did not include a study that only shows remission in thymectomized patients [38], but we included a previous study by the same author that analyzes remission in both groups [26], so there was no duplication of patients. We also excluded patients with thymoma in this study, so in our review, this study [26] has a smaller number of patients. In studies that included patients with pure ocular shape [24, 31], we excluded these patients, reducing the number of cases also in this study. We excluded studies that chose only the best patients for the surgical group [39, 40], and a study where it was not possible to separate the thymomas [41]. Where it was possible to separate the thymomas, these were removed [26–28].

Unfortunately, in this review it was not possible to conduct subgroup analyzes by type of thymectomy, gender, and age, what might elucidate the great heterogeneity found. In the RCT of Wolfe et al. [14], however, both age groups, above 40 years and under 40 years, benefited more from thymectomy than from medical treatment. The same was true for females. In males there was probably no difference due to the small number.

Despite the small number of studies included and the majority of them being case-control, the meta-analysis showed a difference in the outcomes favoring the surgery; RCTs [14, 23] that could not be combined in meta-analysis, also showed the superiority of thymectomy over medical treatment.

This review is evidence for therapeutic decision-making in the treatment of MG at the present time, being one more alternative to guide professionals. Only a small number of the Cochrane collaboration’s systematic reviews support clinical interventions with no need for additional research [42]. May research continue, but we must update our concepts with what we have at the moment.

Almost 80 years ago, thymectomy was first used for the treatment of MG [9]. The recently published RCT by Wolfe et al. [14] took nine years to show the benefit of thymectomy in the treatment of non-thymomatous MG. If we are to wait for other RCTs to accept this treatment along with the clinical treatments employed, instead of treating it as an uncertain or optional intervention, we may take another 80 years to accept an effective surgical treatment for non-thymomatous MG.

## Conclusion

We concluded that thymectomy is effective in the treatment of nonthymomatous MG with remission rates greater than non-surgical treatment. At the moment we need studies that show which subgroups would most benefit from the treatment.

### Study limitations

The main limitation of this review is the fact that only two RCTs were found and all other studies were case-control. Another limitation was the inability to conduct subgroup analyzes.

## Abbreviations

CI: Confidence interval
HR: Hazard ratio
I^2^: Heterogeneity
MG: Myasthenia gravis
MGFA: Myasthenia Gravis Foundation of America
NNT: Number needed to treat
OR: Odds ratio
R/MM: Remission or minimal manifestation
RCT: Randomized clinical trial
